# A Social Media Peer Group Intervention for Mothers to Prevent Obesity and Promote Healthy Growth from Infancy: Development and Pilot Trial

**DOI:** 10.2196/resprot.5276

**Published:** 2016-08-02

**Authors:** Rachel S Gruver, Chanelle T Bishop-Gilyard, Alexandra Lieberman, Marsha Gerdes, Senbagam Virudachalam, Andrew W Suh, Gurpreet K Kalra, Sheela N Magge, Justine Shults, Mark S Schreiner, Thomas J Power, Robert I Berkowitz, Alexander G Fiks

**Affiliations:** ^1^ Division of General Pediatrics The Children's Hospital of Philadelphia Philadelphia, PA United States; ^2^ Department of Child and Adolescent Psychiatry and Behavioral Sciences The Children’s Hospital of Philadelphia Philadelphia, PA United States; ^3^ Department of Psychiatry Perelman School of Medicine University of Pennsylvania Philadelphia, PA United States; ^4^ Department of Pediatrics Perelman School of Medicine University of Pennsylvania Philadelphia, PA United States; ^5^ Division of Gastroenterology, Hepatology and Nutrition The Children’s Hospital of Philadelphia Philadelphia, PA United States; ^6^ Division of Endocrinology and Diabetes, Center for Translational Science Children’s National Health System The George Washington University School of Medicine and Health Sciences Washington, DC United States; ^7^ Department of Biostatistics and Epidemiology Perelman School of Medicine University of Pennsylvania Philadelphia, PA United States; ^8^ Department of Anesthesiology and Critical Care Medicine The Children’s Hospital of Philadelphia Philadelphia, PA United States; ^9^ Division of Developmental and Behavioral Pediatrics The Children’s Hospital of Philadelphia Philadelphia, PA United States

**Keywords:** obesity, social media, behavior change, intervention, internet, peer group, pediatrics, prevention and control, infant, mothers

## Abstract

**Background:**

Evidence increasingly indicates that childhood obesity prevention efforts should begin as early as infancy. However, few interventions meet the needs of families whose infants are at increased obesity risk due to factors including income and maternal body mass index (BMI). Social media peer groups may offer a promising new way to provide these families with the knowledge, strategies, and support they need to adopt obesity prevention behaviors.

**Objective:**

The aim of this study is to develop and pilot test a Facebook-based peer group intervention for mothers, designed to prevent pediatric obesity and promote health beginning in infancy.

**Methods:**

We conducted in-depth semi-structured interviews with 29 mothers of infants and focus groups with 30 pediatric clinicians, to inform the development of a theory-based intervention. We then conducted a single-group pilot trial with 8 mothers to assess its feasibility and acceptability. All participants were recruited offline at pediatric primary care practices. Participants in the pilot trial joined a private Facebook group, moderated by a psychologist, with a weekly video-based curriculum, and also had the option to meet at a face-to-face event. Within the Facebook group, mothers were encouraged to chat, ask questions, and share photos and videos of themselves and babies practicing healthy behaviors. Consistent with the literature on obesity prevention, the curriculum addressed infant feeding, sleep, activity, and maternal well-being. Feasibility was assessed using the frequency and content of group participation by mothers, and acceptability was measured using online surveys and phone interviews.

**Results:**

Based on preferences of mothers interviewed (mean BMI 35 kg/m^2^, all Medicaid-insured, mean age 27, all Black), we designed the intervention to include frequent posts with new information, videos showing parents of infants demonstrating healthy behaviors, and an optional face-to-face meeting. We developed a privacy and safety plan that met the needs of participants as well as the requirements of the local institutional review board (IRB), which included use of a “secret” group and frequent screening of participant posts. Clinicians, 97% (29/30) women and 87% (26/30) pediatricians, preferred no direct involvement in the intervention, but were supportive of their patients’ participation. In our 8-week, single group pilot trial, all participants (mean BMI 35 kg/m^2^, all Medicaid-insured, mean age 28, all Black) viewed every weekly video post, and interacted frequently, with a weekly average of 4.4 posts/comments from each participant. All participant posts were related to parenting topics. Participants initiated conversations about behaviors related to healthy infant growth including solid food introduction, feeding volume, and managing stress. All 8 pilot group participants reported that they found the group helpful and would recommend it to others.

**Conclusions:**

Our methodology was feasible and acceptable to low-income mothers of infants at high risk of obesity, and could be adapted to implement peer groups through social media for underserved populations in varied settings.

**ClinicalTrial:**

ClinicalTrials.gov NCT01977105; https://clinicaltrials.gov/ct2/show/NCT01977105 (Archived by WebCite at http://www.webcitation.org/6iMFfOBat)

## Introduction

Overweight in infancy is common and associated with later obesity and adverse health outcomes [1-4]. According to national data, nearly 10% of infants and toddlers have an elevated weight for recumbent length [5]. This risk increases among those born to women with obesity, families in poverty, and racial/ethnic minorities [1,5-9]. Research suggests that infants with rapid growth during the first 2 years of life are more likely to become obese later in childhood and as adults [4,10,11]. Evidence increasingly indicates that the first 6 months of life are an especially critical time period; growth velocity during the first 4-6 months has been shown to predict obesity at ages 1, 3, 5, 10, and 20 years [2,3,12,13].

In 2011, the Institute of Medicine (IOM) emphasized the need for interventions in early childhood to prevent the subsequent development of obesity [14]. To date, however, traditional obesity prevention strategies (i.e. doctor’s office or home-based parent education) have had mixed results when applied to early childhood, with high levels of treatment engagement typically needed to motivate parent behavior change [15-24]. Furthermore, very few effective interventions have been designed to meet the needs of the low-income, overweight or obese mothers whose infants are at greatest risk. The few that do exist for this population are primarily home visiting programs, which are labor-intensive, making them difficult and often expensive to scale [21-24].

Peer interventions delivered through social media represent a promising alternative to traditional peer interventions, home visiting, or pediatric office-based strategies to promote healthful behaviors and improve outcomes. In pediatrics, peer interventions have been successfully used to provide patients and families with information, support, and problem-solving skills, resulting in improved breastfeeding rates and reduced postpartum depression [25,26]. Peers have also been used to enhance the effectiveness of interventions for populations at high risk of adverse outcomes [22]. However, engaging families at high risk with in-person peer groups can be challenging because of the logistical difficulties they often confront in attending these groups [27].

Social media is a prevalent communication format that is especially well-matched to the delivery of peer interventions. Use is widespread; 90% of online young adults use social media to connect with peers [28]. A growing majority access social media using mobile phones with app capabilities (smartphones that function as computers, have Internet access, and can download apps), now owned by 85% of young adults in the United States. Mobile phones currently serve as the primary source of Internet access for nearly 1 in 5 low-income households, making them a particularly fitting intervention delivery strategy for this population [29]. Mobile phones also allow social media users to interact frequently and at their convenience, a pattern likely to facilitate engagement and delivery of a high “dose” of the intervention. Video content delivered through social media can help to overcome literacy barriers. Since social media is widely and freely accessible, interventions developed using this medium may be more readily disseminated than those requiring the adoption of new technology or frequent, face-to-face interaction.

Consistent with the American Heart Association’s prioritization of social media as a tool to address obesity [30], we developed a Facebook intervention and then tested the feasibility and acceptability of this innovative approach to promote behaviors associated with healthy weight from infancy. Our intervention was designed to address the needs of families with children at highest risk of obesity. Given the potential long-term health benefits of establishing healthy growth in infancy, the need for effective interventions that keep lower-income parents engaged, and the promise of peer interventions delivered via social media, we hypothesized that this approach would be feasible and acceptable to low-income mothers.

## Methods

### Overview

To understand how to best implement prevention-oriented virtual peer groups with low-income mothers at high risk of having obese children, we conducted two different research studies designed to develop, refine, and pilot-test our approach. The Children’s Hospital of Philadelphia (CHOP) Institutional Review Board (IRB) determined that the intervention development study was exempt from review, and approved the pilot study.

### Intervention Development Study

#### Study Design

We conducted in-depth, semi-structured interviews with mothers of infants (subsequently referred to as intervention development interviews), and focus groups with pediatric clinicians. We specified an *a priori* sample size of up to 30 mothers and 30 clinicians since prior research suggests that this number is sufficient to achieve saturation on themes elicited in qualitative interviews [31]. With this number as a guide, data collection and analysis continued iteratively until saturation of themes was reached.

#### Setting and Study Procedures

All participants were recruited from three high-volume, urban, resident teaching primary care practices in the CHOP Pediatric Research Consortium (PeRC), a 2-state practice-based research network. Through rosters generated from the electronic health record (EHR), we identified and then approached potentially eligible mothers at their infant’s primary care visit, where they completed a screening questionnaire for eligibility that assessed criteria not available in the EHR. Consistent with our focus on reaching mothers of infants at high risk of developing obesity [6,7], we enrolled women who were obese with self-reported, pre-pregnancy body mass index (BMI) greater than or equal to 30 kg/m^2^, and had a Medicaid-insured infant, as an indicator of income. Participating mothers were at least 18 years of age and English-speaking with a child up to 1 year old. Clinicians participating in this phase of the study were non-trainees practicing at included sites. All participants provided written informed consent.

Interview and focus group guides were developed that addressed key themes relating to the implementation strategy and content of the intervention. In interviews, mothers were also asked for their opinions on sample intervention curriculum content, delivered through brief videos. Interviews were audiotaped, transcribed, and analyzed using QSR NVIVO10 software (QSR, Cambridge, MA). We used content analysis [32,33] to identify themes that emerged regarding the curriculum, implementation strategy, and outcome measurement protocols for the planned intervention. The constant comparative method, in which newly collected data are compared with categories that have emerged from previously collected data, was used throughout the data analysis to identify emerging themes to inform the planned intervention [34]. As data collection progressed, the research team discussed emerging themes, and iteratively updated the interview guide to refine our results. A coding scheme and coding dictionary were developed. The analysis was conducted by 2 coders; double coding was used on a majority (76%, 22/29) of the transcripts to establish consistency of the coding scheme. If differences in coding arose, the coders discussed them and reached a consensus. Representative verbatim comments were selected for presentation.

### Single Group Pilot Trial

#### Study Procedures

Incorporating findings from the intervention development study, we subsequently conducted an 8-week single-group pilot trial of an actual Facebook peer group in order to assess the feasibility and acceptability of the virtual peer group format. Eight overweight or obese mothers of Medicaid-insured newborns (<1 month) were recruited at PeRC sites using the same methods and inclusion criteria described in the intervention development study above. Further eligibility screening criteria for participation in the pilot trial included owning a mobile phone with a data plan, and the ability to take photos and videos using the phone. In addition, to focus on mothers whose needs could be addressed by the intervention, mothers were excluded from the pilot trial if they screened positive for clinical depression on the Patient Health Questionnaire-9 [35], had not received prenatal care, delivered before 37 weeks gestation, or if they had gestational diabetes, a multiple gestation pregnancy, or an infant hospitalized in the neonatal intensive care unit for 1 week or longer.

Study participation involved Facebook group activities for 8 weeks and an optional in-person meeting prior to the start of the Facebook group intervention. Participants also completed an online questionnaire at baseline and at study end, and an in-depth, semi-structured phone interview regarding their satisfaction and experiences with the group. Online surveys were completed using Research Electronic Data Capture (REDCap) hosted at CHOP. REDCap is a secure, Web-based application for data collection that provides an intuitive interface for validated data entry [36]. Baseline study measures included demographic characteristics, household food security measured using a validated 2-item questionnaire from the US Household Food Security Survey Module [37,38], and health literacy measured using the Newest Vital Sign Questionnaire [39]. In order to assess the feasibility of measuring outcomes, mothers’ beliefs regarding infant feeding were measured on both the pre- and post-intervention surveys using relevant items from the Infant Feeding Style Questionnaire [40]. Surveys also included multiple choice and open-ended items measuring the acceptability of the intervention. We used interview and survey responses along with the content and rate of participant activity (posts/comments, “likes,” and “seen by” counts) to identify successful aspects of the peer group and general considerations for the implementation of virtual peer groups, including the selection of measures of impact.

#### Initial Peer Group Design

Participants were informed that they would participate in an 8-week Facebook group focused on healthy infant growth. All were assigned to a single peer group, facilitated by a psychologist with expertise in obesity treatment. The group began with an in-person baby shower at which participants could meet other group members and the facilitator in person. Following this event, and in contrast to many previously published interventions in which social media was only a small component [27,41-43], the entire intervention occurred online, in a Facebook group accessible only to study staff and invited participants. The group was set up as “secret,” Facebook’s maximum privacy setting, which restricts visibility of the group to current members. Group activities included viewing weekly educational videos posted to the group that featured mothers and infants (including many from the same community) modeling behaviors and addressing topics related to healthy infant growth. Specifically, the curriculum addressed infant feeding, sleep, activity, and maternal well-being. Participating mothers then posted their own photos, videos and experiences, provided feedback on posts by other group members and received feedback from peers and the group moderator.

This intervention design was based on Social Learning Theory [44] which emphasizes the importance of observing models in preparation for performing a behavior, then receiving positive feedback after practicing the behavior. In this case, behavioral models were provided by parents in the curriculum videos and by the group facilitator, as well as participants’ own photos and videos. The facilitator provided positive feedback to participants whose posts demonstrated healthy behaviors by directly providing “likes” and comments, and encouraging other participants to do the same. In this way, the moderator role was central to this theory-based intervention.

Curriculum content was developed locally based on results from intervention development interviews with mothers and clinician focus groups, and national guidelines for pediatric prevention and health promotion [45]. Content included weekly modules that consisted of a short video, as well as a brief written summary of key points from the video (which was posted to the Facebook group both as a text post and as a downloadable PDF handout). Shorter posts throughout the week included infant “fun facts” or health tips, which, when relevant, included hyperlinks to outside resources.

As the study involved participants using their personal mobile phones to access the group, each received a US $50 monthly stipend for 2 months to offset the approximate cost of their phone data plan. Participants were told that in order to be eligible for the stipend, they needed to post or comment in the Facebook group at least once; they were encouraged to log in at least weekly, but, beyond that basic guidance, were told that they could participate in the group as much or as little as they wanted. Participants who did not access the group for over 2 weeks received a private Facebook message with a reminder from the group facilitator.

#### Development of the Human Subjects Plan

In order to ensure that our Facebook intervention sufficiently protected human participants, we consulted extensively with the CHOP IRB throughout the intervention development process. The IRB concluded that the intervention met the regulatory definition of minimal risk, as the activities involved posed no greater risk than those encountered in during daily life [46]. Though the risks to participants were minimal, the study safety plan employed several strategies to further minimize risk. First, access to the Facebook group was limited to individuals who had consented to participate, and the group was moderated. Second, the facilitator or study staff reviewed all posts for appropriateness of tone and content (e.g., not offensive or critical of other group members). New posts were delayed until after they had been reviewed, and posts that failed to meet the terms of the group were excluded.

From an IRB perspective, breaches of confidentiality represented the most significant risk for participants. To mitigate this risk, clear rules were established and conveyed to prospective participants as part of the informed consent process, with ground rules posted on the group page (information posted by others should be treated as confidential; others’ identities should not be revealed outside the group). The consent (Multimedia Appendix 1) made clear that the confidentiality of information posted in the group could not be guaranteed.

To complement the perspectives of the IRB, both intervention development interview participants as well as pilot group members were asked to comment on the human subjects approaches proposed for the pilot group study.

## Results

### Study Population

A total of 29 mothers of children up to 1 year old participated in the intervention development interviews and survey; participants had a mean BMI of 35 kg/m^2^, mean age of 27 years, and were all of Black race (Table 1), though race/ethnicity were not inclusion criteria for the study. As an indicator of socioeconomic status, only Medicaid-enrolled families were eligible for the study. Over half were at risk of household food insecurity [37,38]. Nearly all were current, frequent Facebook users. In addition, 30 clinicians participated in focus groups (29 women; 26 pediatricians and 4 nurse practitioners; 24 White, 3 Black, 3 Asian; mean 14 years post-training). In the pilot trial of the intervention, 8 mothers participated with a mean BMI of 35 kg/m^2^, mean age 28, and all Black (Table 1). Mothers all reported an annual income of less than US $15,000, and rates of food insecurity were similar to those in the intervention development interview group. All had existing Facebook accounts at the time of enrollment, though this was not a requirement for eligibility.

**Table 1:** Characteristics of participating mothers in each study, measured at enrollment.

Characteristics		Interviews (N=29), n (%)	Pilot group (N=8), n (%)
Sex			
	Female	29 (100)^a^	8 (100)^b^
Age, years			
	18-25	13 (45)	4 (44)
	26-30	12 (41)	2 (25)
	≥31	4 (13)	2 (25)
Race/Ethnicity^c^			
	Black	29 (100)	8 (100)
	White	1 (3)	1 (13)
	American Indian/Alaska Native	0 (0)	1 (13)
	Hispanic/Latino	2 (%)	0 (0)
Highest education level completed			
	High school or less	16 (55)	4 (50)
	Some college/associate degree	10 (35)	3 (38)
	Bachelors or professional degree	3 (10)	1 (13)
Annual income			
	< $10,000	N/A	6 (75)
	$10,000-$14,999	N/A	2 (25)
Number of children			
	1	11 (38)	2 (25)
	2-3	14 (48)	3 (38)
	≥ 4	4 (14)	3 (38)
Weight category			
	Overweight (25 kg/m^2^ ≤ BMI < 30 kg/m^2^)	N/A	2 (25)^b^
	Obese (BMI ≥ 30 kg/m^2^)	29 (100)^a^	6 (75)^b^
Household food insecurity			
	At risk	15 (52)	4 (50)
Technology use			
	Have a Facebook account	25 (86)	8 (100)
	Own a mobile phone	25 (86)	8 (100)^b^
	Pay for mobile phone data plan	19 (66)	8 (100)^b^
Breastfeeding			
	Breastfeeding only	N/A	0 (0)
	Formula only	N/A	5 (63)
	Both breast and formula	N/A	3 (38)
Health literacy (Newest Vital Sign)			
	High likelihood or possibility of limited literacy	N/A	5 (63)
	Adequate literacy	N/A	3 (38)

^a^Female sex and obesity (BMI≥ 30kg/m^2^) were inclusion criteria for the intervention development interviews.

^b^Female sex, overweight or obesity (BMI≥ 25kg/m^2^), and mobile phone/data plan ownership were inclusion criteria for the single-group pilot trial.

^c^Participants were instructed to select all applicable categories; hence the totals are more than 100%.

### Implementation Strategies

#### Overview

Mothers and clinicians in the intervention development study commented on several dimensions of the intervention implementation strategy. Their responses are outlined below, followed by a description of how this information was used to develop the intervention. We then present the results and participant feedback related to study implementation from the pilot trial.

#### Web-Based Versus Face-To-Face Activities

In intervention development interviews, participants were asked how they would feel about participating in an online group with other mothers they had not met in person. Responses and levels of concern were quite varied. While many (59%, 17/29 mothers) were unconcerned (“That sounds perfectly fine”), for a few (10%, 3/29) it would strongly affect their willingness to participate (“[If] I don’t know who they are, I won’t share information with you, you know?”). Several others (31%, 9/29) had perspectives between these extremes:

I don’t know – A little antsy, but I’d manage. I wouldn’t go into so much detail about my life… I’d keep it at a certain level.

After several mothers suggested that even one in-person event for the group would assuage their concerns about not knowing the other participants, a question about this was added to the interview guide. Of the respondents asked, 75% (15/20) said that they would like to meet in person. As one participant put it:

It’s better to know them more direct than indirectly. You don’t really know them as well on Facebook than face to face.

Others commented that a face-to-face event would help keep them engaged and interested.

Based on this input, the pilot peer group began with an in-person event, with a baby shower theme. Of the participants, 38% (3/8) attended along with the group facilitator. One participant later commented:

I met [the facilitator] at the get together. It was good to meet her first and that way I was comfortable enough to talk in the group.

Overall, participants who attended the in-person event had similar rates of participation compared to those who did not attend (median of 23 posts/comments for all participants over the course of the intervention in both groups).

#### Engagement Strategies

In intervention development interviews, mothers discussing the role of a peer group in their lives anticipated that the primary benefits or reasons to participate would be (1) learning new things about parenting (97%, 28/29); and (2) peer support and interaction with other mothers (93%, 27/29). When asked specifically what would be most helpful in keeping them engaged, responses reflected similar priorities. One participant replied:

To know each mother’s experience, to know what they went through, what’s been on their mind….That would keep me involved.

Many respondents (76%, 22/29) expressed a desire or need for support in this context, and described connecting with other mothers as a way to learn and potentially adopt better parenting behaviors. For example, in the words of one mother:

The support [would be the most helpful thing]. Just knowing that you have somebody like your peers that’s there that can answer questions for you… [M]others telling you this is what they did and it worked for them.

Another commented, "You would get to meet new people, learn different methods people are using to raise their kids.” A first-time mother anticipated “asking questions from mothers that already had kids.

One respondent stated that if she’d had the opportunity to participate in such a peer group,

I would have been more confident when [my baby] came home… taking care of her, knowing what to do, because everyone has different experience and you learn from other people.

Another engagement strategy that mothers suggested was to keep the group active with frequent posts and new, up-to-date information about parenting. Both the frequency and quality of information were considered important. Mothers requested “New topics, not the same thing over and over again, learn new things,” and “Just making sure I have all the up to date, like, maybe articles and stuff like that on children and babies.” Based on this feedback, groups included frequent posts of new information that augmented the curriculum.

#### Use of Video

When mothers were shown sample videos created for the group, several (24%, 7/29) noted that it was helpful to see actual parents and children in the videos, particularly when they demonstrated behaviors or activities. One commented:

I connected with everybody [in the video] because they all were interacting with their babies, it’s not like they were just on there by themselves saying, “Oh this is what I do”. They was always holding their babies and show them what they do.

Mothers suggested that watching these modeled behaviors would trigger action. One said:

[A mother in the video] sung a song I’ve never heard of. I’d probably sing that with my son.

#### Privacy

The vast majority of intervention development interview respondents (93%, 27/29), none of whom had ever participated in a similar intervention, reported that they would feel comfortable participating in an intervention that used a “secret” Facebook group (a privacy setting which limits group visibility and access to those invited by the moderator). For more than half (55%, 16/29), this comfort level was due specifically to the “secret” privacy setting. The mothers interviewed liked that it would be “just moms and nobody else,” and “not letting the whole world see it,” so that “whatever I said would just be between a certain amount of people.” Another commented that “[Facebook is] pretty good with the secret groups.”

Some mothers (21%, 6/29) felt that privacy was not a concern because they did not intend to share any information that they perceived as too personal. For example, one participant stated she would not be worried about privacy because “If I’m not comfortable saying it to your face, I’m not gonna post it.” Several clarified that they did not see parenting as a very private or sensitive topic: “I mean, as far as parenting, that’s fine pretty much, that stuff I’d feel comfortable with sharing.”

In the abbreviated pilot trial, the “secret” group setting was used. No participant reported any breach of privacy or concern about privacy, either during or after the study.

#### Role of the Group Facilitator

When asked about the ideal role of a group facilitator, many intervention development interview respondents (55%, 16/29) stated that they would want her to provide information or advice: “Just help answer questions, feeding, breastfeed, solid foods, I guess the leader would know all of that.” Seven specified explicitly (and others implied) that they would want the facilitator to be an experienced mother, who could draw upon her own experience to provide guidance to the group. “A hands on mom has most likely been through stuff, probably can give us realistic…ideas and other ways and methods.” Others said that they would want the facilitator to provide emotional support, keep group discussions on track, manage conflicts, or provide resources for low-income mothers. The group facilitator in the pilot trial was a PhD-level psychologist who was also an experienced mother with young children. She frequently posted in the group (14.4 posts per week on average), modeling healthy behaviors and providing information (in 46%, 53/115 facilitator posts), and offering emotional support and encouragement (17%, 19/115 posts), as well as guiding group discussion.

#### Monitoring Plan

Our monitoring plan was intended to prevent any harm to participants caused by inappropriate or offensive posts by other group members. This plan was described to mothers during intervention development interviews; all agreed that having all posts screened by the group leader before appearing on the group page would be both reasonable and helpful: “That’s great, you know, some people might post things other people may not want to see.” However, one noted that quick approval would be necessary to prevent the approval process from interfering with participation:

I think that would be fine, as long as they approve them fast. You don’t wanna have to wait days for your post to be posted…. When you post something usually it’s on there right away. I’d give y’all a couple of hours, maybe.

In the pilot trial, the group facilitator and one additional member of the research staff screened and approved posts regularly throughout each day, including weekends and at night, accessing the group from mobile devices to facilitate this process. Participants did not report any problems or concerns with this approach. Group members respected ground rules established to guide interactions. In fact, during the 2-month trial, only one comment was not approved for posting by the facilitator. This comment expressed a participant’s frustration with others in the group for not responding quickly to a question she had posted; the facilitator messaged the participant privately to discuss the post. The participant continued in the group with no further issues.

#### Involvement of Pediatric Clinicians

We asked both mothers and clinicians about the best way, if any, to involve the pediatric practice in the intervention. Mothers were asked whether they would want any of their information from the group shared with their child’s pediatrician. Most were relatively comfortable with this: “I wouldn’t mind. If they wanna share something with the doctor that would be fine, because I know that would be kept confidential.”

When asked about information sharing in the other direction, from pediatricians to the group leader, responses were more varied. Some mothers were comfortable with “good” information being shared:

[I would want shared] that my baby is healthy, well taken care of, eating right, growing. All the good things.

However, for information about problems or concerns, “It would depend on what problem,” and many participants would want to approve that information on a case-by-case basis before it was shared.

In focus groups, clinicians expressed enthusiasm for the intervention, and were willing to receive and share information with the intervention team on an as-needed basis. However, clinicians preferred no direct involvement in the intervention. The intervention was designed as suggested with no direct pediatrician participation.

### Feasibility of a Prevention-Oriented Peer Group

#### Enrollment and Access

Of the 10 eligible participants approached, 8 participants enrolled in the pilot study and successfully joined the Facebook group. One additional mother began enrollment and provided informed consent, but did not complete the enrollment survey and was unable to be contacted further; based on the limited information obtained, her characteristics were similar to the participants who enrolled successfully. Almost all of the participants reported accessing the group primarily via mobile phone; 2 used both phones and computers to access the group, and 1 reported using a tablet and a computer, but not a mobile phone. All were successfully able to view the group and create their own posts.

All 8 participants successfully completed online surveys at baseline and study end, including items assessing infant feeding beliefs and behaviors [40]. In interviews, participants reported that they understood all survey items and had no questions or concerns. Outcome data are not reported here as the objective of this study was to assess the feasibility and acceptability of our outcome measurement plan.

#### Engagement

**Figure 1:**
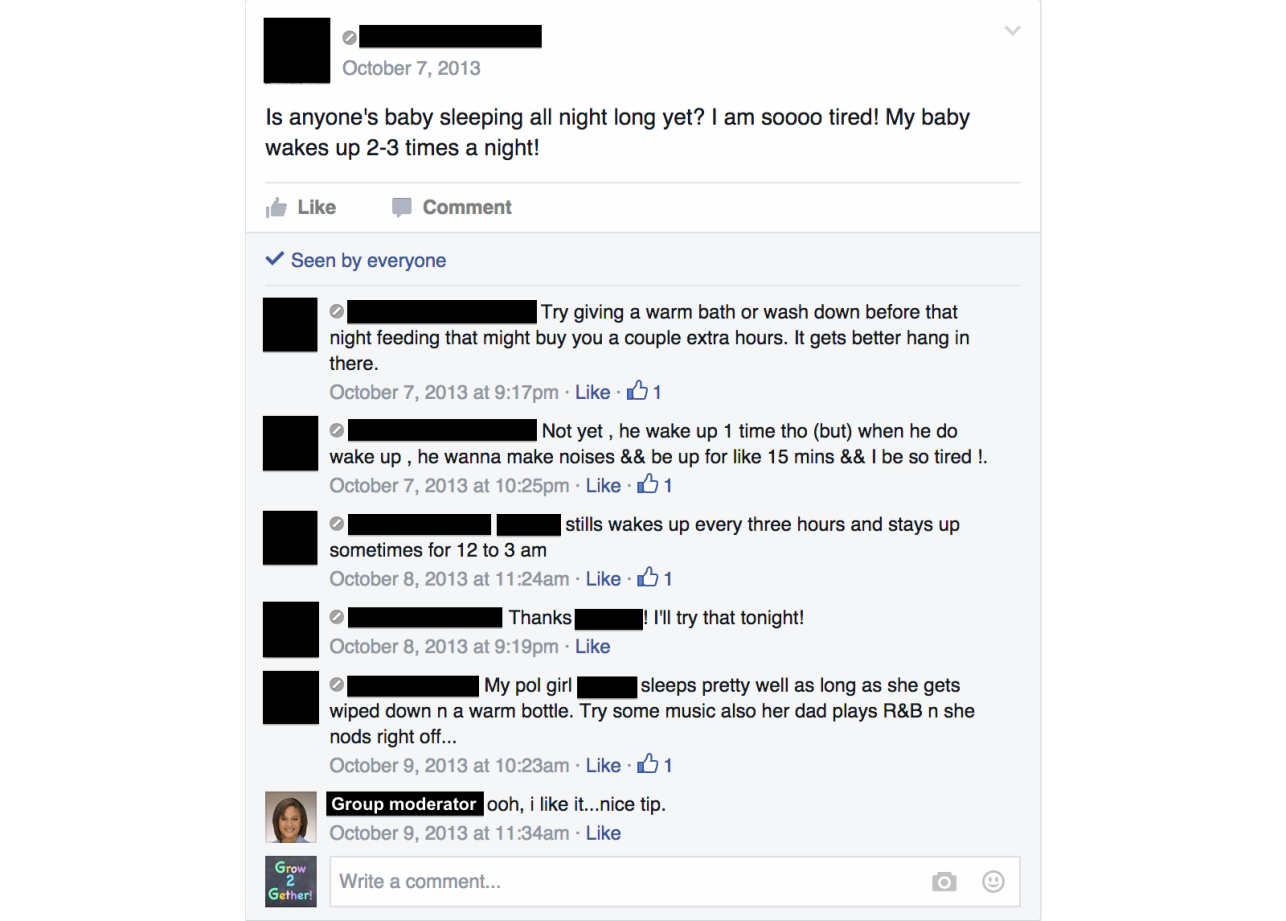
Participant-initiated conversation about infant sleep (participant information redacted, profile image shown for facilitator only).

**Figure 2:**
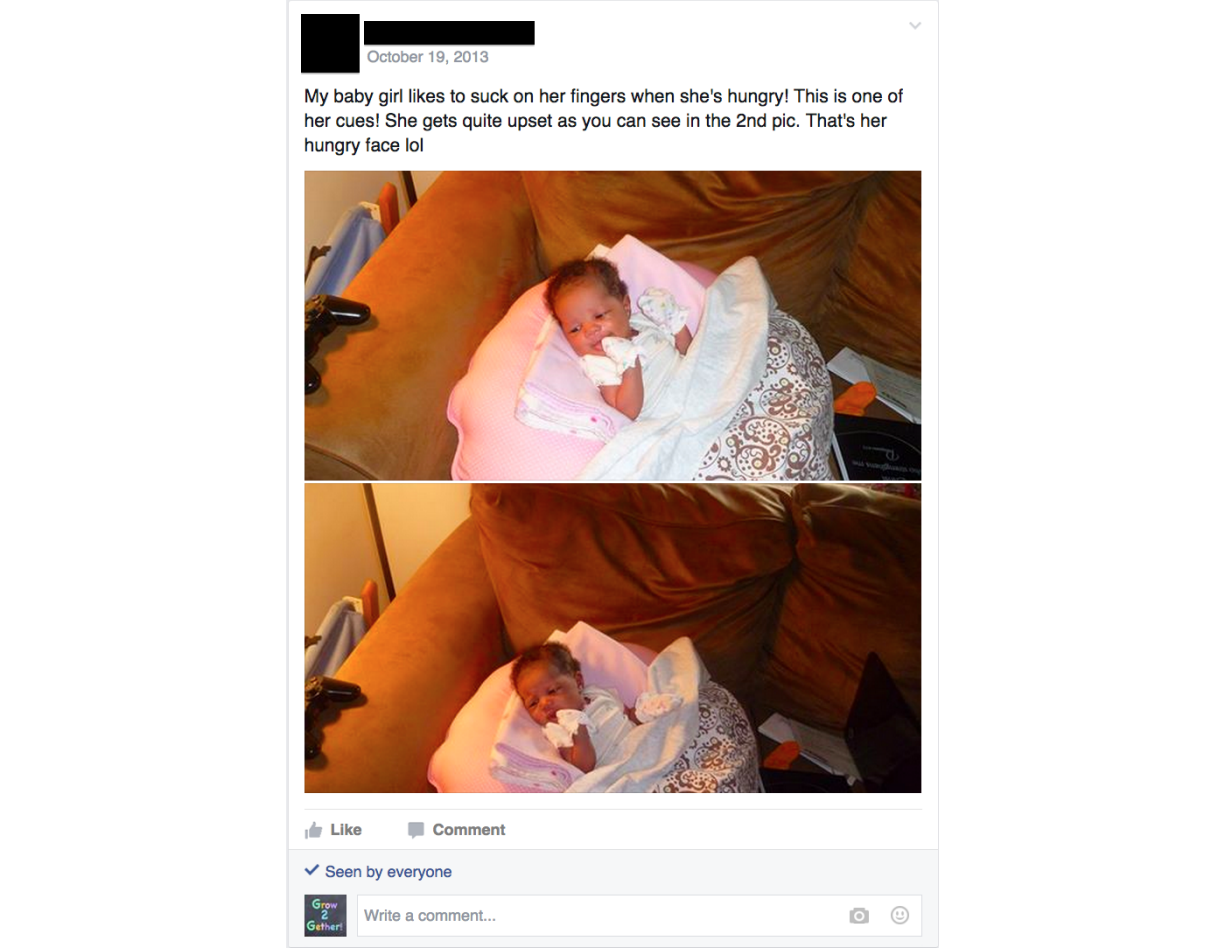
Participant post showing infant hunger cues. This photo was approved for publication by the participant.

The Facebook format created a record of all group activities, facilitating analysis. All active participants viewed all of the weekly videos, and the group averaged 7 posts and 6 “likes” daily. Of the participant posts, 91.8% (257/280) were text, 6.4% (18/280) were photos, and 1.8% (5/280) were videos. All participant posts were related to parenting topics. Participants initiated conversations about behaviors related to infant growth, including topics such as solid food introduction, sleep schedules (Figure 1), feeding volume, and managing stress. Consistent with the curriculum, mothers’ photos and videos demonstrated responses to infant cues (Figure 2), soothing behaviors, infant play, and sleep routines. In this small sample, participation did not vary between first-time and experienced mothers.

Participants were successful in supporting one another in a virtual group format. The theme of peer support emerged strongly in surveys and interviews of the mothers in the pilot group. One participant commented that, “I felt like part of a group when I was participating and I felt like I helped someone along the way and vice versa.” Reflecting how close participants in virtual peer group could become, one wrote, “…all of us as MOMS came together n [*sic*] got along so well without us even knowing each other.” Another mother added that “The group wasn’t really like a group of strangers talking, it was more like sisters helping each other out.”

As further evidence of the peer connections established in the group, 5 of the participants reported at the end of the pilot study that they had become Facebook friends with at least one of the other group members. They also reported interactions with group members that went on outside of the group itself:

We exchanged numbers and friended and messaged each other on regular Facebook. We post on each other’s walls and message each other.

Another explained, “After the group was over, we all wanted to stay in contact.” Participants reported that topics of discussion outside of the group generally mirrored those in the group, such as feeding and sleep schedules, although some conversations were more specific and personal, such as 2 mothers whose children were both hospitalized who messaged one another about the experience.

#### Group Impact

All 8 participants reported that they had gained knowledge from the group. In surveys, when asked if participating in the group had helped them, one commented, “I learned some new techniques for the baby,” while another wrote, “Yes, new information about babies’ feeding playtime activities and more, because a lot of things have changed since I had my last child 13 years ago.” Others even indicated that the benefits went beyond simply learning, to shifts in attitude: “It helped me grow as a parent to different ideas.”

On the survey, mothers were also asked if they had changed anything they did, or planned to, based on the group. More than half (63%, 5/8) reported that they had, primarily making adjustments to their child’s feeding and sleep schedules (both of which were behavioral targets of the intervention).

### Acceptability of a Prevention-Oriented Peer Group

The pilot group participants found the Facebook group highly acceptable. All (100%, 8/8) active participants agreed with the statements, “I would recommend this program,” and “The program was helpful.” When asked, “What did you think of the Facebook group?” responses were consistently positive, and focused on peer connections (88%, 7/8) and sharing of information as the most helpful aspects: “I think that it was amazing that I got to talk to other moms that were experiencing the same thing that I was.”

It was nice to share with other new mothers. Even though this is baby number five for me it was nice to share some of the things I have learned over the years.

I think it was a great group overall. I learned some good tips and got some good advice.

While half of the participants stated that they would not want to change anything about the group, the others had the following 2 recurring suggestions: (1) to add more participants to the peer group (suggested by 3 participants), and (2) to have the 2-month long group run for a longer period of time (suggested by 4 participants).

All participants interviewed reported that they found the curriculum very helpful; all preferred information in video format (vs electronic documents or information posted directly to the Facebook group as a text comment). One elaborated:

I liked the videos because it was actual parents showing you how to do things. Rather than having it written out.

Another explained:

The videos [were best], because I was able to see it. I’m a visual kind of person.

Although videos were preferred, 2 participants commented that they appreciated having the information presented in multiple formats: “The videos were helpful, I liked it better with the steps written out under the video post.”

[The facilitator] was good. Because she…posted the information in multiple ways, written out and in the videos.

All pilot group participants had only positive feedback about the group facilitator’s role. In interviews, one participant commented that, “[She] was great, very informative, gave great advice, answered all my questions, answered questions very quickly.” Another said:

I loved her, she was very cool. Big sister type thing. I felt like I could talk to her about anything.

Others specifically appreciated her level of involvement in the group:

Some leaders give you stuff to do and then are absent. She was around and attentive, anything you posted she would comment on….

## Discussion

### Principal Findings

Input from low-income mothers and the clinicians who care for their children guided our development of a prevention-oriented Facebook peer group intervention that was feasible, acceptable, and engaging to participants. Based on mothers’ preferences, we designed the intervention to include frequent posts with new information, videos showing parents demonstrating behaviors associated with obesity prevention, and a face-to-face group event prior to starting the Facebook group intervention. We developed a privacy and safety plan that met the needs of participants as well as the requirements of the local IRB, including use of a “secret” group and frequent screening of participant posts. In our 8-week pilot group, all participants viewed every weekly video post and interacted frequently, with each participant posting or commenting an average of 4.4 times per week. All 8 pilot group participants reported that they found the group helpful and would recommend it to others.

### Comparison with Prior Work

Although our pilot group was small, rates of participation and satisfaction with the intervention were remarkably high compared with other social media-based peer group interventions in the literature. For example, an 8-week physical activity intervention delivered via Facebook group and text messages to 29 African-American women had 0.2 posts per participant per week, 22 times lower than in our group [47]. Rates of participation were nearly as low in studies of other prevention-oriented Facebook groups, including those focused on smoking, physical activity, and postpartum weight loss [48-50].

There are many potential contributing factors to the high levels of engagement in our group. Though other social media peer groups designed for demographically similar groups have not been as successful in maintaining engagement [27,47], the population participating in our intervention may have been particularly well-suited to a Facebook-based peer group, since almost all were existing frequent Facebook users and were new mothers interested in sharing photos of their children. In addition, in contrast to other interventions that split up participant interactions across multiple other platforms including text messages, phone calls, and/or other websites [27,47,51,52], Facebook was the sole delivery method of our intervention curriculum. Another unique aspect of the intervention was its focus on infant health, which may have been a more compelling topic for mothers than the maternal health outcomes targeted elsewhere [53], such as physical activity, nutrition and/or weight [27,49]. The role of the group facilitator, who, consistent with Social Learning Theory [44], provided positive reinforcement for participation and posted frequently in the group herself, may have also encouraged participants to post more often. It is possible that the US $50 monthly incentive to cover the cost of mobile phone data for active participants may have motivated some of the activity in the group. However, participants only had to post or comment in the group once to be considered “active,” and all participants posted much more frequently (35 times on average).

Participants told us that protecting their privacy and safety was essential to the success of the group. Having a “secret” group setting that restricted group access to members was especially important for many participants to feel comfortable sharing information about themselves and their children. This finding has been supported by similar research; several Facebook-based interventions in the literature also used a “private” or “secret” group setting [41,54,55]. In one Facebook intervention study, a smoking cessation group was changed to a private group setting after participants expressed concerns about confidentiality [48]. Although our findings apply specifically to Facebook’s current features and settings, the privacy needs of participants will certainly remain relevant to peer group success even as social media platforms and features change. In establishing safety measures, we worked carefully to balance the preferences and concerns of participants, the requirements of the IRB, and the logistical realities of the study team. For example, our procedure for monitoring posts was recommended by the IRB, and approved by mothers with the caveat that posts be screened and approved quickly. In order to meet both of these needs, we determined that more than one member of the study team would be needed to continuously screen posts, and mobile phones should be used by the team to facilitate access. Our approach may provide a model for those seeking to develop similar interventions that are acceptable to the IRB, and safe and engaging for participants.

One question that remains to be answered is the role of face-to-face meetings as a component of social media interventions. Other recent Facebook-based interventions have incorporated one or more in-person meetings [41,56], and this approach has been recommended by others as a way to increase engagement [47]. In our case, the idea was generated by mothers in intervention development interviews, and was preferred over an online chat or phone meeting by 75% (15/20) of the mothers we asked. The mothers in the pilot trial who attended the meeting reported that it was a helpful introduction that made them feel more comfortable posting to the group. This raises the possibility that social media peer groups may be more engaging for some participants if circumstances permit a face-to-face meeting first. However, this does not appear to be the case for all participants, since only 38% (3/8) of the mothers in our group actually attended the event, and those who attended participated in the peer group at similar rates as those who did not. Furthermore, members of some online groups may be geographically distributed too broadly to meet in one location, or, especially in the case of youth and those with low income, may lack transportation [27]. Further research is needed to better understand the necessity and ideal role of offline meetings as part of social media peer group interventions.

### Limitations

The primary limitation of this study is its small sample size, particularly in the pilot trial. This study was designed to develop a Facebook peer intervention; a randomized controlled trial of a longer intervention curriculum, with more participants and multiple peer groups, is currently under way. In addition, the mothers participating in this study represent a specific and narrow sample of women, whose children are at increased risk of obesity due to maternal BMI, race, and income. While this group represents a population in particular need of support, their experiences and opinions of a social media-based peer intervention may differ from those of other populations. In the pilot trial, the population was further limited to mobile phone owners; 2 mothers were excluded from participating for this reason. The majority of mothers approached did own a mobile phone, however, as do a growing majority of adults in the United States, including those with low income [29].

Furthermore, this study tested intervention delivery using Facebook groups, and it is unclear as social media evolves how the intervention may adapt to changing Facebook features or translate to other platforms. Still, the principle of delivering a moderated peer group to low-income mothers using familiar technology will likely remain relevant. Finally, this study assessed the feasibility of measuring the intended behavioral and health outcomes of the intervention. However, future studies, such as the ongoing pilot trial, are needed in order to actually understand the impact that this social media peer group intervention may have on participants’ knowledge, attitudes, behaviors, and health.

### Conclusions

This intervention provides a model for the design and use of private social media groups as a platform to deliver peer-based interventions to change health behaviors. Our results indicate that such groups are both feasible and acceptable, even among extremely low-income populations whose children are at high risk for obesity and other adverse health outcomes.

## Appendix

Multimedia Appendix 1 Pilot trial consent form.

## Abbreviations

BMI: body mass index
CHOP: The Children’s Hospital of Philadelphia
EHR: electronic health record
IRB: institutional review board
PeRC: Pediatric Research Consortium
REDCap: Research Electronic Data Capture
