# Supplementary material for: A Social Media Peer Group Intervention for Mothers to Prevent Obesity and Promote Healthy Growth from Infancy: Development and Pilot Trial
Source: JMIR Res Protoc. 2016 Aug 2;5(3):e159. doi: 10.2196/resprot.5276 (PMC4987492; doi:10.2196/resprot.5276)
Supplement: Multimedia Appendix 1 [file resprot_v5i3e159_app1.pdf]

Informed Consent Form and HIPAA Authorization

Study Title: Abbreviated Pilot Trial of A Peer-Based Social Media Intervention to Promote Healthy Growth During Infancy

Version Date: September 23, 2013

Consent Name: Abbreviated Pilot (1C) Main Study Consent

Principal Investigator: Alexander Fiks, MD Telephone: (267) 426-2304

You and your child may be eligible to take part in a research study. This form gives you important information about the study. It describes the purpose of this research study, and the risks and possible benefits of participating.

If there is anything you do not understand, please ask questions. Please take your time. You do not have to take part in this study if you do not want to. If you take part, you can leave the study at any time.

The word "we" means the study doctor and other research staff.

Why are you being asked to take part in this study?

You are being asked to take part in this research study because you have completed our screening survey and meet all of the conditions for enrollment in the study, including being age 18 or older, able to speak, read and write in English, and owning a smartphone.

What is the purpose of this research study?

We are conducting a research study on a new program for mothers. The purpose of the program is to help mothers help their infants to grow and develop in a healthy way.

The purpose of this study is to test part of the program with a small group of mothers.

How many people will take part?

About 10 mothers and their infants will take part in this study.

What is involved in the study?

How long will you be in this study?

If you agree to take part, your participation will last for about 2 months. It will involve 2 study visits (including today's visit), and participation in a small peer group of mothers in a private Facebook group.

What are the study procedures?

The study involves the following tests and procedures.

Questionnaire: The study involves completing some questionnaires about yourself and your family. We will also ask you to complete questionnaires about your satisfaction with the program and any suggestions you may have.

Infant weight and length measurement: A study staff member will measure your infant's weight and length at this visit and at his/her 2-month primary care visit, or we will record the measurements taken by primary care staff from your child's medical record.

Baby Shower Party: This party will be held soon after you join the study, and will give you a chance to meet the group leader and the other moms in your group face to face. You may attend in person, or join us via video chat.

Facebook group: You will be asked to participate in a private Facebook group. An expert from the Children's Hospital of Philadelphia (CHOP) will lead the groups. Activities in the Facebook group will involve watching short videos about taking care of your baby, then posting photos or videos of you and your baby demonstrating what you have learned. These activities will occur about once a week (about 8 activities over the course of the study). Group members can use the Facebook group to comment on each other's photos and videos and give one another support and advice. You will also participate in in at least one phone call with the leader of your group.

Appointment reminders: You will receive text message reminders for your child's primary care doctors appointments at CHOP Care Network sites.

Chart review: The study staff may access your child's medical chart for information related to the study, such as weight and length, contact information or scheduled appointments.

Visit Schedule

The table below provides a brief description of the purpose and approximate duration of each study visit.

**Visit**	**Purpose**	**Main Procedures**	**Duration**
Visit 1: Today	Screening and enrollment	Questionnaires, infant weight and length measurement (*if you do not have time to finish questionnaires at this visit, you can finish them online, over the phone, or at the baby shower*)	20 - 40 minutes
Baby Shower Party	Meet your group	Introductory games and activities	1-2 hours
Online activities (taking place over 2 months)	*Group activities*	*Online photo/video activities and discussion every week*<br>*Short satisfaction questionnaire for each activity*	*8, 45 minute activities*<br>*(Total: 6 hours)*
Visit 2: Infant's 2-month visit	End of Study	Questionnaires, infant weight and length measurement	1 hour

What are the risks of this study?

Taking part in a research study involves inconveniences and risks. If you have any questions about any of the possible risks listed below, you should talk to the study doctor or your regular doctor.

While in this study, you are at risk for the following:

Risks associated with interviews and questionnaires: You may feel uncomfortable while completing the surveys or the interview, because of the personal nature some of the questions about you and your child. Once you start the surveys or once the interview starts, you can skip any question or stop filling out the survey or participating in the interview at any time and for any reason.

Risks associated with chart review and questionnaire data collection: As with any study involving collection of data, there is the possibility of breach of confidentiality of data. Every precaution will be taken to secure your personal information to ensure confidentiality.
At the time of participation, each participant will be assigned a study identification number. This number will be used on data collection forms, questionnaires and in the database instead of names and other private information. A separate list will be maintained that will link each participant's name to the study identification number for future reference and communication.

Risks associated with participation in the Facebook group:

We cannot guarantee the confidentiality of information that you share with your peer group within the private Facebook group or in person. Therefore, we ask that you not share information that is very private or sensitive in that setting. Information on group ground rules and confidentiality will be provided on the group's Facebook page.

Another ground rule of group participation will be respect for others in the group. The study team will screen posts, and monitor the group to remove any inappropriate or harmful comments. However, it is possible that others may post statements or images that are upsetting or that make you uncomfortable, either independently or in response to your own posts. In such cases, the group moderator will work with you and the other participants to resolve the problem or conflict.

Additionally, you should be aware that the following conditions apply to all Facebook users:

- Facebook will have access to your IP address and location.
- Facebook will have the right to use the photos and videos you post. This right ends when you delete the photos/videos, or your account. However, if you share the photos and videos with others on Facebook, Facebook will be able to access and use that content until all others have deleted it as well.

More detailed information on Facebook, privacy, and private group participation will be provided to you by the study team in a separate brochure.

Are there any benefits to taking part in this study?

There may be no direct benefit to you for taking part in this study, though some mothers may benefit from receiving information about infant health and/or their own health, and/or from receiving text message appointment reminders. Information obtained from this study will be used to understand whether this program works, and to improve the program as needed.

Specifically, this information may help researchers understand how to better provide health information using social media, and how to better work with mothers to promote healthy infant growth.

Do you need to give your consent in order to participate?

If you decide to participate in this study, you must sign this form. A copy will be given to you to keep as a record. Please consider the study time commitments and responsibilities as a research subject when making your decision about participating in this study. You will need to follow the study doctor's instructions and keep all study appointments. You will need to participate in your peer group as directed.

What happens if you decide not to take part in this study?

Participation in this study is voluntary. You do not have to take part in order for your child to receive care at CHOP.

If you decide not to take part or if you change your mind later there will be no penalties or loss of any benefits to which you are otherwise entitled.

Can you stop your participation in the study early?

You can stop being in the study at any time. You do not have to give a reason.

If you withdraw from the study, you will no longer receive monthly payments towards the cost of your phone's data and texting plan.

Can the study doctor take you out of the study early?

The study doctor may take you off of the study if you cannot meet all the requirements of the study.

What choices do you have other than this study?

There are options for you other than this study including:

- Not participating in this study and receiving regular infant primary care at CHOP or elsewhere.

What about privacy, authorization for use of Personal Health Information (PHI) and confidentiality?

As part of this research, health information about you and your child will be collected. This will include information from medical records, questionnaires, measurements, and your peer group's Facebook page. We will do our best to keep your personal information private and confidential. However, we cannot guarantee absolute confidentiality. Your personal information may be disclosed if required by law.

The results of this study may be shown at meetings and published in journals to inform other doctors and health professionals. We will keep your identity private in any publication or presentation.

Several people and organizations may review or receive your identifiable information. They will need this information to conduct the research, to assure the quality of the data, or to analyze the data or samples. These groups include:

- Members of the research team and other authorized staff at CHOP
- People from agencies and organizations that perform independent accreditation and/or oversight of research; such as the Department of Health and Human Services, Office for Human Research Protections.

In addition, the following organizations may review or receive certain identifiable information:

- Public health authorities that are required by law to receive information for the prevention or control of disease, injury or disability (child abuse/neglect, etc.)
- If you provide us with your primary care doctor's contact information, we may share limited information with them if there is an urgent concern about your health (for example, severe depression)
- Facebook will have access to any information you post within the private group.

By law, CHOP is required to protect your health information. The research staff will only allow access to your health information to the groups listed above. By signing this document, you are authorizing CHOP to use and/or release your health information for this research. Some of the organizations listed above may not be required to protect your information under Federal privacy laws. If permitted by law, they may be allowed to share it with others without your permission.

The identifiable information from this study will be kept for at least 6 years or until the study is completed, whichever is longer. Your permission to use and share the information and data from this study will continue until the research study ends and will not expire. Researchers continue to analyze data for many years and it is not possible to know when they will be completely done.

Can you change your mind about the use of personal information?

You may change your mind and withdraw your permission to use and disclose your health information at any time. To take back your permission, you must tell the investigator in writing.

Dr. Alexander Fiks
The Children's Hospital of Philadelphia
3535 Market St.
Philadelphia, PA 19104

In the letter, state that you changed your mind and do not want any more of your health information collected. The personal information that has been collected already will be

used if necessary for the research. No new information will be collected. If you withdraw your permission to use your personal health information, you will be withdrawn from the study.

Financial Information

While you are in this study, the cost of your usual medical care – procedures, medications and doctor visits – will continue to be billed to you or your insurance.

Will there be any additional costs?

To participate in this study, you must have a smartphone with a data and text messaging plan. You must continue to pay for your plan while you are enrolled in the study.

All other costs of participating in this study will be covered by the study. There are no additional costs to you or your insurance.

Will you be paid for taking part in this study?

- You will be paid $50 each month that you participate in the study (for approximately 2 months; a total of $100) to cover the cost of your cell phone's data and text messaging plan, which you will need to participate.
- You will receive $20 when you complete study questionnaires at your infant's 2-month primary care visit.
- If you attend the baby shower party, you will receive a small gift worth approximately $10.

Payments will be provided in the form of CHOP incentive cards (bank cards). The bank providing the card will have access to your name, address and phone number.

Who is funding this research study?

This study is funded by the CHOP Healthy Weight Program, which received financial support for research from the American Beverage Association Foundation for a Healthy America.

What if you have questions about the study?

If you have questions about the study, call the study doctor, Dr. Alexander Fiks at 267-426-2304. You may also talk to your child's doctor if you have questions or concerns.

The Institutional Review Board (IRB) at The Children's Hospital of Philadelphia has reviewed and approved this study. The IRB looks at research studies like these and makes sure research subjects' rights and welfare are protected. If you have questions about your rights or if you have a complaint, you can call the IRB Office at 215-590-2830.

Consent for Use of Data for Future Research

As part of the study, we will collect questionnaire information, measurements of your infant's weight and length, and information on program Facebook participation. We may wish to use this information in a future study about this program or other Facebook-based health programs. The information will be given a unique code and will not include information that can identify you or your child. Information that can identify you or your child may be kept permanently on a secure, password-protected computer at CHOP. Only the study doctors and those working with them on this study will be able to see information that can identify you.

If you leave the study, you can ask to have the data collected about you removed. You can also ask us to remove information that identifies you from the data.

Please indicate whether you will allow your questionnaire responses, your child's measurements, and your Facebook group participation data to be used for future research by putting your initials next to one of the following choices:

_____ (initials) My <u>information and my child's measurements</u> may be used for this study only.

_____ (initials) My <u>information and my child's measurements</u> may be used for other future research studies. If the data are shared outside of CHOP, no identifiable information will be included.

Consent to Take Part in this Research Study and Authorization to Use and Disclose Health Information for the Research

The research study and consent form have been explained to you by:

Person Obtaining Consent	Signature of Person Obtaining Consent
	Date

By signing this form, you are indicating that you have had your questions answered, you agree to take part in this research study and you are legally authorized to consent to your child's participation. You are also agreeing to let CHOP use and share your and your child's health information as explained above. If you don't agree to the collection, use and sharing of your and your child's health information, you and your child cannot participate in this study. **NOTE:** *A foster parent is not legally authorized to consent for a foster child's participation.*

Name of Subject (mother)

Signature of Subject (18 years or older)	Date

Child Participation:

Name of Subject (child)

Name of Authorized Representative<br>(if different than participating mother)	Relation to subject:<br>☐ Parent ☐ Legal Guardian
Signature of Authorized Representative	Date
